# Predicting patients' arrival to the Emergency Department UKMMC

**DOI:** 10.1186/1472-6963-12-S1-P5

**Published:** 2012-11-21

**Authors:** Noriza Majid, Sharifa Ezat WP, Saperi Sulong, Zuraidah Che Man

**Affiliations:** 1 School of Mathematical Sciences, Faculty of Science and Technology, UKM, Selangor, Malaysia; 2 Department of Community Health, Faculty of Medical, UKM, Kuala Lumpur, Malaysia; 3 Department of Accident and Emergency, Faculty of Medical, UKM, Kuala Lumpur, Malaysia

## Objectives

To model and predict daily patients’ arrival to the Emergency Department (ED) based on preceding year data using parametric fitting methods.

## Methods

Daily patients’ arrival starting from the year of 2005 to 2009 to the ED of UKM Medical Centre was studied. The patients’ arrival patterns were described. Poisson and Negative Binomial models that are commonly used in modeling frequency were selected to represent the number of patient seeking treatment at ED per day. Maximum likelihood method is used in estimating the parameters for both distributions. Models accuracy were assessed by comparing the predicted arrivals that obtained from the proposed models and observed arrival using goodness of fit test.

## Results

The best model to predict the patients’ arrival to the ED is based on the results of p-p plots, the Schwarz-Bayesian criteria, the maximum likelihood function and chi-squared test. Results from the analysis shows that for five consecutive years, the patients’ arrival to the ED at UKM Medical Centre follows the Negative Binomial distribution with an average of 192 patients a day.

## Conclusion

This study confirms that the number of arrival of the patients explains the daily demand for the A&E services. It is very important for the department to understand the pattern of patients’ arrival in order to help the hospital strategize and optimize its resources.

